# The Listsize Capacity of the Gaussian Channel with Decoder Assistance

**DOI:** 10.3390/e24010029

**Published:** 2021-12-24

**Authors:** Amos Lapidoth, Yiming Yan

**Affiliations:** Signal and Information Processing Laboratory, ETH Zurich, 8092 Zurich, Switzerland; yan@isi.ee.ethz.ch

**Keywords:** bit pipe, cutoff rate, decoder assistance, Gaussian channel, helper, listsize capacity

## Abstract

The listsize capacity is computed for the Gaussian channel with a helper that—cognizant of the channel-noise sequence but not of the transmitted message—provides the decoder with a rate-limited description of said sequence. This capacity is shown to equal the sum of the cutoff rate of the Gaussian channel without help and the rate of help. In particular, zero-rate help raises the listsize capacity from zero to the cutoff rate. This is achieved by having the helper provide the decoder with a sufficiently fine quantization of the normalized squared Euclidean norm of the noise sequence.

## 1. Introduction

**Figure 1:**
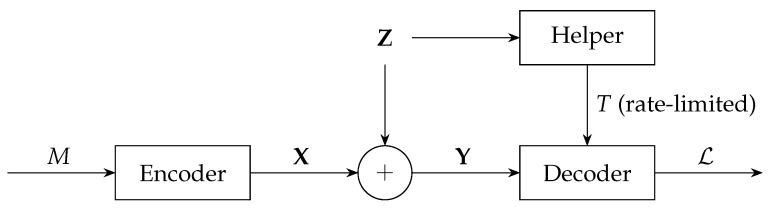
Gaussian channel with decoder assistance.

The order-ρ listsize capacity $C_{list}^{\left(\rho \right)}$ of a channel is the supremum of the coding rates for which there exist codes guaranteeing the large-blocklength convergence to one of the ρ-th moment of the cardinality of the list of messages that, given the received output sequence, have positive a posteriori probability. It is zero for the Gaussian channel because, on this channel, no codeword is ruled out by any received sequence so said list contains all the messages. Here we derive this capacity for the Gaussian channel with a helper that observes the noise sequence and describes it to the decoder using a rate-limited noise-free bit pipe; see Figure 1.

We show that the listsize capacity $C_{list}^{\left(\rho \right)}\left(R_{h}\right)$ is then the sum of bit-pipe’s rate $R_{h}$ and the order-ρ cutoff rate $R_{cutoff}\left(\rho \right)$ of the Gaussian channel without a helper

(1) $$\begin{array}{ccc} C_{list}^{\left(\rho \right)}\left(R_{h}\right) & = & R_{cutoff}\left(\rho \right)+R_{h}. \end{array}$$

The latter’s definition is similar to that of the listsize capacity, but with the list now comprising only those messages that are a posteriori at least as likely as the transmitted one. As we shall see, for the Gaussian channel with average power P, noise-variance N, and corresponding signal-to-noise ratio (SNR) A≜P/N,

(2) $$\begin{array}{ccc} R_{cutoff}\left(\rho \right) & = & R_{0}\left(\rho \right), \end{array}$$

where

(3) $$\begin{array}{ccc} R_{0}\left(\rho \right) & = & \frac{1}{2}\ln\frac{1}{2}\left(1+\frac{A}{1+\rho }+\sqrt{{\left(1-\frac{A}{1+\rho }\right)}^{2}+\frac{4A}{{\left(1+\rho \right)}^{2}}}\right)\\ & & +\frac{1+\rho }{2\rho }\frac{1}{2}\left(1+\frac{A}{1+\rho }-\sqrt{{\left(1-\frac{A}{1+\rho }\right)}^{2}+\frac{4A}{{\left(1+\rho \right)}^{2}}}\right)\\ & & +\frac{1}{2\rho }\ln\frac{1}{2}\left(1-\frac{A}{1+\rho }+\sqrt{{\left(1-\frac{A}{1+\rho }\right)}^{2}+\frac{4A}{{\left(1+\rho \right)}^{2}}}\right) \end{array}$$

(in nats) is a function that plays a prominent role in the analysis of the Reliability Function of said channel (Section 7.4 in [1]), [2]. That analysis does not, however, carry over directly to our setting because it deals with error exponents and not lists.

It is interesting to note that (1) also holds when the help rate $R_{h}$ is zero: the number of help bits required to increase the listsize capacity from zero to $R_{cutoff}\left(\rho \right)$ is sublinear in the blocklength. In fact, as we shall see, all it takes is a sufficiently fine quantization of the normalized squared Euclidean norm of the noise sequence.

The relation (1) is reminiscent of the analogous result on the erasures-only capacity $C_{\text{e-o}}\left(R_{h}\right)$ of the Gaussian channel with a rate-$R_{h}$ helper (Remark 10 in [3]), namely, that

(4) $$\begin{array}{ccc} C_{\text{e-o}}\left(R_{h}\right) & = & C+R_{h}, \end{array}$$

where *C* denotes the Shannon capacity of the Gaussian channel (without help) (Theorem 9.1.1 in [4]), and $C_{\text{e-o}}\left(R_{h}\right)$ is the erasures-only capacity, which is defined like $C_{list}^{\left(\rho \right)}\left(R_{h}\right)$ but with the requirement on the ρ-th moment of the list replaced by the requirement that the list be of size 1 with probability tending to one. (The Gaussian erasures-only capacity with a helper is given by the RHS of (4) irrespective of whether the assistance is provided to the encoder or decoder.) The latter result in turn is reminiscent of the analogous result on the Shannon capacity with a helper $C(R_{h})$ [5,6,7,8]

(5) $$\begin{array}{ccc} C(R_{h}) & = & C+R_{h}. \end{array}$$

In proving (1), we shall focus on the “direct part,” i.e., that the right-hand side (RHS) of (1) is achievable. The “converse,” that no rate exceeding the RHS of (1) is achievable, is omitted because it follows directly from (Remark 4 in [3]): There it is shown that this is true even if, given the received sequence and the provided help, the list contains only a *subset* of the messages that are of positive a posteriori probability, namely, those that are a posteriori at least as likely as the transmitted message.

The listsize capacity is relevant, for example, when the message set corresponds to tasks [9] and the transmitted message corresponds to one that must be performed by the decoder with absolute certainty. To ensure this, the decoder must perform all the tasks in the list of tasks that are not ruled out by the received sequence. (In addition to the transmitted task, other tasks need not but may be performed.) The ρ-th moment of the list’s size then measures the receiver’s average effort.

Results on the listsize capacity and the erasures-only capacity of general discrete memoryless channels (DMCs) in the absence of help are scarce. Noteworthy exceptions are the results of Pinsker and Sheverdjaev [10], Csiszár and Narayan [11], and Telatar [12], that provide sufficient conditions for the erasures-only capacity to equal the Shannon capacity and for the listsize capacity to equal the cutoff rate. Asymptotic results on the erasures-only capacity in the low-noise regime can be found in [13,14]. Once noiseless feedback is introduced, the problems become more tractable [15,16,17].

The rest of the paper is organized as follows. Section 2 describes our set-up and presents the main result. Section 3 contains some classical and some new observations regarding Gallager’s $E_{0}$ function and its modification. Section 4 derives the cutoff rate of the Gaussian channel without help and proves (2). Section 5 describes and analyzes a coding scheme that proves the direct part of (1).

## 2. The Main Result

A power-P blocklength-*n* encoder $f^{\left(n\right)}$ for a message set M is a mapping

(6) $$f^{\left(n\right)}:M\to R^{n}$$

that maps each message m∈M to an *n*-tuple $f^{\left(n\right)}\left(m\right)$ whose Euclidean norm $∥f^{\left(n\right)}\left(m\right)∥$ satisfies

(7) $$∥f^{\left(n\right)}{\left(m\right)∥}^{2}\leq nP,\;m\in M.$$

We sometimes use $x_{m}$ to denote $f^{\left(n\right)}\left(m\right)$, and $x_{m,k}$ to denote the *k*-th component of $x_{m}$, so

(8) $$f^{\left(n\right)}\left(m\right)=x_{m}=\left(x_{m,1},\ldots ,x_{m,n}\right).$$

The encoder is said to be of rate *R* if the cardinality of M is $e^{nR}$, in which case we often assume that $M=\{1,\cdots ,e^{nR}\}$. (We ignore the fact that $e^{nR}$ need not be an integer; this issue washes out in the large-*n* asymptotics we study.)

When a message m∈M is sent over the discrete-time additive Gaussian noise channel using the encoder $f^{\left(n\right)}$, the channel produces the random vector $Y\in R^{n}$ whose *k*-th component $Y_{k}$ is

(9) $$Y_{k}=x_{m,k}+Z_{k},\;k=1,\ldots ,n,$$

where $\left\{Z_{k}\right\}$ are independent and identically distributed (IID) zero-mean Gaussians of variance N. We assume that N is positive and use w(y|x) to denote the density of the channel’s output when its input is *x*, i.e., the mean-*x* variance-N Gaussian density

(10) $$w\left(y|x\right)=\frac{1}{\sqrt{2\pi N}}e^{-\frac{{\left(y-x\right)}^{2}}{2N}},\;x,y\in R,$$

which we extend to *n*-tuples in a memoryless fashion:

(11) $$w\left(y|x\right)=\prod_{k=1}^{n}w\left(y_{k}|x_{k}\right),\;x,y\in R^{n}.$$

For convenience, we define

(12) $$A=\frac{P}{N}.$$

Given an output sequence y and a message *m*, we define the “at-least-as-likely list”

(13) $$L\left(m,y\right)=\left\{m^{\prime }\in M:w\left(y|x_{m^{\prime }}\right)\geq w\left(y|x_{m}\right)\right\}.$$

Assuming, as we do, that the messages are a priori equally likely, this list comprises the messages that, given the output sequence y, are a posteriori at least as likely as *m*.

If a message *M*, drawn equiprobably from M, is transmitted over the channel with a resulting received sequence Y, then the cardinality of the at-least-as-likely list is a random positive integer, and we denote its ρ-th moment $E\left[{\left|L\left(M,Y\right)\right|}^{\rho }\right]$:

(14) $$E\left[{\left|L\left(M,Y\right)\right|}^{\rho }\right]=\frac{1}{\left|M\right|}\sum_{m\in M}\int w(y|x_{m}{)\;|L\left(m,y\right)|}^{\rho }d\nu \left(y\right),$$

where ν(·) denotes the Lebesgue measure on $R^{n}$.

For a given ρ>0, we define the order-ρ cutoff rate $R_{cutoff}\left(\rho \right)$ as the supremum of the rates *R* for which there exists a sequence of rate-*R* power-P blocklength-*n* encoders $\left\{f^{\left(n\right)}\right\}$ satisfying

(15) $$\lim_{n\to \infty }E\left[{\left|L\left(M,Y\right)\right|}^{\rho }\right]=1.$$

**Theorem** **1.** *The order-ρ cutoff rate $R_{cutoff}\left(\rho \right)$ of the additive Gaussian noise channel equals $R_{0}\left(\rho \right)$ of* (3).

**Proof.**  See Section 4. □

A $T_{n}$-valued description of the noise sequence $Z=(Z_{1},\ldots ,Z_{n})$ is a mapping

(16) $$\phi^{\left(n\right)}:R^{n}\to T_{n}$$

with the understanding that $\phi^{\left(n\right)}\left(Z\right)$, which we denote *T*, is the description of Z. We say that a sequence $\left\{\phi^{\left(n\right)}\right\}$ of descriptions is of rate $R_{h}$ (nats) if

(17) $$\lim_{n\to \infty }\frac{1}{n}\;\ln\left|T_{n}\right|=R_{h}.$$

Suppose now that, in addition to the received sequence Y, the receiver is also presented with the description $T=\phi^{\left(n\right)}\left(Z\right)$ of the noise, and that, based on the two, it forms the “remotely-plausible list” L(Y,T) comprising the messages that have positive a posteriori probability given the two:

(18) $$L\left(y,t\right)=\left\{m\in M:\phi^{\left(n\right)}\left(y-x_{m}\right)=t\right\}.$$

Given ρ>0, the listsize capacity $C_{list}^{\left(\rho \right)}\left(R_{h}\right)$ with rate-$R_{h}$ decoder assistance is the supremum of the rates *R* for which there exists a sequence of rate-*R* power-P blocklength-*n* encoders $\left\{f^{\left(n\right)}\right\}$ and a sequence $\left\{\phi^{\left(n\right)}\right\}$ of descriptions of rate $R_{h}$ such that

(19) $$\lim_{n\to \infty }E\left[{\left|L\left(Y,\phi^{\left(n\right)}\left(Z\right)\right)\right|}^{\rho }\right]=1.$$

**Theorem** **2.** *On the Gaussian channel, the listsize capacity with rate-$R_{h}$ decoder assistance $C_{list}^{\left(\rho \right)}\left(R_{h}\right)$ is given by*

(20) $$\begin{array}{ccc} C_{list}^{\left(\rho \right)}\left(R_{h}\right) & = & R_{cutoff}\left(\rho \right)+R_{h} \end{array}$$

*where $R_{cutoff}\left(\rho \right)$ is the order-ρ cutoff rate of the channel (without assistance) as given in* (2) *and* (3).

**Proof.**  The “converse,” that (19) cannot be achieved when the rate exceeds the RHS of (20), follows from (Remark 4 in [3]). The “direct part,” describing a coding scheme that achieves (19) with rates approaching the RHS of (20), is proved in Section 5. □

## 3. Preliminaries

Given ρ≥0 and any probability measure *Q* on R, Gallager’s $E_{0}$ function for our channel is defined as [1]

(21) $$E_{0}\left(\rho ,Q\right)=-\ln\int_{y\in R}{\left(\int_{x\in R}w{\left(y|x\right)}^{\frac{1}{1+\rho }}dQ\left(x\right)\right)}^{1+\rho }d\nu (y),$$

where ν(·) is now the Lebesgue measure on R. The result of maximizing $E_{0}\left(\rho ,Q\right)$ over all *Q* under which $E[X^{2}]\leq P$, is denoted $E_{0}^{*}\left(\rho \right)$:

(22) $$\begin{array}{ccc} E_{0}^{*}\left(\rho \right) & = & \underset{Q:\int x^{2}dQ\left(x\right)\leq P}{\sup}E_{0}\left(\rho ,Q\right). \end{array}$$

The multi-letter extension of $E_{0}$ is

(23) $$E_{0}^{\left(n\right)}\left(\rho ,Q^{\left(n\right)}\right)=-\frac{1}{n}\ln\int_{y\in R^{n}}{\left(\int_{x\in R^{n}}w{\left(y|x\right)}^{\frac{1}{1+\rho }}dQ^{\left(n\right)}\left(x\right)\right)}^{1+\rho }d\nu \left(y\right),$$

where $Q^{\left(n\right)}$ is a probability measure on $R^{n}$; the integrals are over $R^{n}$; the channel w(y|x) is defined in (11). Similarly,

(24) $$\begin{array}{ccc} E_{0}^{(n),*}\left[n\right]\left(\rho \right) & = & \underset{Q^{\left(n\right)}:\int {∥x∥}^{2}dQ^{\left(n\right)}\left(x\right)\leq nP}{\sup}E_{0}^{\left(n\right)}\left(\rho ,Q^{\left(n\right)}\right). \end{array}$$

Given probability measures $Q^{\left(m\right)}$ on $R^{m}$ and $Q^{\left(n\right)}$ on $R^{n}$ that satisfy the power constraints $E[∥X{∥}^{2}]\leq mP$ and $E[∥X{∥}^{2}]\leq nP$ respectively, the product measure $Q^{\left(m\right)}\times Q^{\left(n\right)}$ on $R^{m+n}$ satisfies the power constraint $E[∥X{∥}^{2}]\leq \left(m+n\right)P$ and

(25) $$\left(m+n\right)\;E_{0}^{\left(m+n\right)}\left(\rho ,Q^{\left(m\right)}\times Q^{\left(n\right)}\right)=m\;E_{0}^{\left(m\right)}\left(\rho ,Q^{\left(m\right)}\right)+n\;E_{0}^{\left(n\right)}\left(\rho ,Q^{\left(n\right)}\right)$$

because

(m+n)E0(m+n)ρ,Q(m)×Q(n)(26)=−ln∫y∈Rm+n∫x∈Rm+nw(y|x)11+ρdQ(m)×Q(n)(x)1+ρdν(y)(27)=mE0(m)(ρ,Q(m))+nE0(n)(ρ,Q(n)).

The sequence $\left\{n\;E_{0}^{(n),*}\left(\rho \right)\right\}$ is thus superadditive, and Feket’s Subadditive lemma implies that $\left\{E_{0}^{(n),*}\left(\rho \right)\right\}$ converges to its supremum:

(28) $$\lim_{n\to \infty }E_{0}^{(n),*}\left(\rho \right)=\underset{n}{\sup}E_{0}^{(n),*}\left(\rho \right).$$

We shall later see (cf. (55) ahead) that

(29) $$\frac{1}{\rho }\;\underset{n}{\sup}E_{0}^{(n),*}\left(\rho \right)=R_{0}\left(\rho \right),$$

where $R_{0}\left(\rho \right)$ is defined in (3).

We shall also need Gallager’s modified $E_{0}$ function. To highlight its relation to the unmodified function, which is quite general, we shall use g(x) for $x^{2}$ and g(x) for ${∥x∥}^{2}$. We shall also replace P with Γ.

Given some ρ≥0, some probability distribution *Q* on R under which E[g(X)]≤Γ, and some r≥0, the modified Gallager’s $E_{0}$ function $E_{0,m}\left(\rho ,Q,r\right)$ is defined as

(30) $$E_{0,m}\left(\rho ,Q,r\right)=-\ln\int_{y\in R}{\left(\int_{x\in R}e^{r(g(x)-\Gamma )}\;w{\left(y|x\right)}^{\frac{1}{1+\rho }}dQ\left(x\right)\right)}^{1+\rho }d\nu \left(y\right).$$

We shall also be interested in the maximum of $E_{0,m}\left(\rho ,Q,r\right)$ over both *Q* and *r*. We distinguish between two cases depending on whether E[g(X)]≤Γ holds strictly or not. In the former case we only allow *r* to be zero, whereas in the latter case it can be any non-negative number. We thus define

(31) $$E_{0,m}^{*}\left(\rho ,Q\right)=\left\{\begin{array}{cc} \sup_{r\geq 0}E_{0,m}\left(\rho ,Q,r\right), & \mathrm{if}\;\int g(x)dQ(x)=\Gamma ,\\ E_{0}\left(\rho ,Q\right), & \mathrm{if}\;\int g(x)dQ(x)<\Gamma , \end{array}\right.$$

and

(32) $$E_{0,m}^{**}\left(\rho \right)=\underset{Q:\int g(x)dQ(x)\leq \Gamma }{\sup}E_{0,m}^{*}\left(\rho ,Q\right).$$

The next proposition provides a lower bound on $\lim E_{0}^{(n),*}\left(\rho \right)$.

**Proposition** **1.** *Any probability distribution Q on R under which g(X) is of finite second moment and of expectation* Γ *provides the lower bound*

(33) $$\lim_{n\to \infty }E_{0}^{(n),*}\left(\rho \right)\geq E_{0,m}^{*}\left(\rho ,Q\right).$$

**Proof.**  Let *Q* be any input distributions *Q* under which g(X) is of finite second moment and E[g(X)]=Γ. For each n∈N, let $Q^{\left(n\right)}$ be the conditional distribution of the *n*-fold product distribution $Q^{\times n}$ given the event $\left\{X\in A_{n}\right\}$, where

(34) $$A_{n}=\left\{x\in R^{n}:n\Gamma -\delta <g\left(x\right)\leq n\Gamma \right\}$$

where δ>0 is some positive constant. Thus, for every Borel measurable subset B of $R^{n}$,

(35) $$Q^{\left(n\right)}\left(B\right)=\frac{1}{\mu }\;Q^{\times n}\left(B\cap A_{n}\right),\;B\in B\left(R^{n}\right)$$

with

(36) $$\begin{array}{c} \mu =Q^{\times n}\left(A_{n}\right). \end{array}$$

For any r≥0, we can upper-bound the Radon–Nykodim derivative of $Q^{\left(n\right)}$ with respect to product distribution $Q^{\times n}$ as follows:

(37)dQ(n)dQ×n=1μI{x∈An}(38)≤1μer(g(x)−nΓ+δ)(39)=1μerδer(g(x)−nΓ)

where I{statement} equals 1 if the statement is true and else 0. Using this bound on the Radon–Nykodim derivative we obtain:

(40)E0(n)(ρ,Q(n))=−1nln∫y∈Rn∫x∈Rnw(y|x)11+ρdQ(n)(x)1+ρdν(y)(41)≥−1nln∫y∈Rn∫x∈Rnw(y|x)11+ρ·1μerδer(g(x)−nΓ)dQ×n(x)1+ρdν(y)(42)=−1+ρnlnerδμ−ln∫y∈R∫x∈Rer(g(x)−Γ)w(y|x)11+ρdQ(x)1+ρdν(y)(43)=−1+ρnlnerδμ+E0,m(ρ,Q,r).

By the Central Limit Theorem, μ tends to 1/2 as *n* tends to infinity, so (43) implies that

(44) $$\begin{array}{c} \underset{n\to \infty }{lim inf}\frac{1}{n}E_{0}^{\left(n\right)}\left(\rho ,Q^{\left(n\right)}\right)\geq E_{0,m}\left(\rho ,Q,r\right). \end{array}$$

Taking the supremum of the RHS over all r≥0, establishes that

(45) $$\begin{array}{c} \underset{n\to \infty }{lim inf}\frac{1}{n}E_{0}^{\left(n\right)}\left(\rho ,Q^{\left(n\right)}\right)\geq E_{0,m}^{*}\left(\rho ,Q\right) \end{array}$$

and hence, by (24), proves (33). □

We next turn to upper-bounding $\lim E_{0}^{(n),*}\left(\rho \right)$.

**Proposition** **2.** *If the probability distribution $Q^{\left(n\right)}$ on $R^{n}$ is such that E[g(X)]≤nΓ, and if $f_{R}$ is any density on R, then*

(46) $$\begin{array}{ccc} E_{0}^{\left(n\right)}\left(\rho ,Q^{\left(n\right)}\right) & \leq & \underset{P:\int g(x)dP(x)\leq \Gamma }{\sup}-\left(1+\rho \right)\int_{x\in R}\ln\left(\int_{y\in R}w{\left(y|x\right)}^{\frac{1}{1+\rho }}f_{R}{\left(y\right)}^{\frac{\rho }{1+\rho }}dy\right)dP\left(x\right) \end{array}$$

*and, consequently,*

(47) $$\begin{array}{ccc} \lim_{n\to \infty }E_{0}^{(n),*}\left(\rho \right) & \leq & \underset{P:\int g(x)dP(x)\leq \Gamma }{\sup}-\left(1+\rho \right)\int_{x\in R}\ln\left(\int_{y\in R}w{\left(y|x\right)}^{\frac{1}{1+\rho }}f_{R}{\left(y\right)}^{\frac{\rho }{1+\rho }}dy\right)dP\left(x\right). \end{array}$$

**Proof.**  The proof is based on Proposition 2 in [18], which implies that for every density $f_{R}^{\left(n\right)}$ on $R^{n}$ and any probability measure $Q^{\left(n\right)}$ on $R^{n}$,

(48) $$n\;E_{0}^{\left(n\right)}\left(\rho ,Q^{\left(n\right)}\right)\leq -\left(1+\rho \right)\int_{x\in R^{n}}\ln\left(\int_{y\in R^{n}}w{\left(y|x\right)}^{\frac{1}{1+\rho }}\;f_{R}^{\left(n\right)}{\left(y\right)}^{\frac{\rho }{1+\rho }}dy\right)dQ^{\left(n\right)}\left(x\right).$$

Applying this inequality to the product density

(49) $$f_{R}^{\left(n\right)}\left(y\right)=\prod_{i=1}^{n}f_{R}\left(y_{i}\right),$$

where $f_{R}$ is a density on R, and using the product form of the channel (11), we obtain that for any density $f_{R}$ on R

(50)E0(n)(ρ,Q(n))≤−1n(1+ρ)∑i=1n∫xi∈Rln∫y∈Rw(y|xi)11+ρfR(y)ρ1+ρdydQi(n)(xi)(51)=−(1+ρ)∫x∈Rln∫y∈Rw(y|x)11+ρfR(y)ρ1+ρdydQ¯(x),

where $Q_{i}^{\left(n\right)}$ is the *i*-th marginal of $Q^{\left(n\right)}$, and $\bar{Q}$ is the probability measure on R defined by

(52) $$\bar{Q}=\frac{1}{n}\sum_{i=1}^{n}Q_{i}^{\left(n\right)}.$$

Observe that if E[g(X)]≤nΓ under $Q^{\left(n\right)}$, then E[g(X)]≤Γ under $\bar{Q}$. This observation and (51) establish (46). Since (46) holds for all *n*, (47) must also hold. □

## 4. The Cutoff Rate of the Gaussian Channel

In this section, we prove Theorem 1. Since scaling the output does not change the cutoff rate, we will assume WLOG that the noise variance is 1 and the transmit power is A; see (12). Thus,

(53) $$w\left(y|x\right)=\frac{1}{\sqrt{2\pi }}e^{-\frac{{\left(y-x\right)}^{2}}{2}},\;x,y\in R,$$

and each codeword $x_{m}$ satisfies

(54) $$\begin{array}{c} ∥x_{m}{∥}^{2}\leq nA. \end{array}$$

### 4.1. Computing $\lim E_{0}^{(n),*}\left(\rho \right)$

Here we shall establish that on the Gaussian channel (53)

(55) $$\lim_{n\to \infty }E_{0}^{(n),*}\left(\rho \right)=\rho R_{0}\left(\rho \right)=E_{0,m}^{*}\left(\rho ,Q_{G}\right),$$

where $R_{0}\left(\rho \right)$ is defined in (3), and $Q_{G}$ is the zero-mean variance-A Gaussian distribution. To this end, we shall derive matching upper and lower bounds on the limit. We begin with the former.

#### 4.1.1. Upper-Bounding $\lim E_{0}^{(n),*}\left(\rho \right)$

We show that on the channel (10)

(56) $$\lim_{n\to \infty }E_{0}^{(n),*}\left(\rho \right)\leq \rho R_{0}\left(\rho \right).$$

The proof is based on Proposition 2 with the density $f_{R}$ corresponding to a centered Gaussian of variance $\sigma^{2}$, where

(57) $$\begin{array}{c} \sigma^{2}=\frac{A}{(1+\rho )\beta }+1 \end{array}$$

and

(58) $$\begin{array}{c} \beta =\frac{1}{2}\left(1-\frac{A}{1+\rho }+\sqrt{{\left(1-\frac{A}{1+\rho }\right)}^{2}+\frac{4A}{{\left(1+\rho \right)}^{2}}}\right). \end{array}$$

Evaluating the RHS of (47) for this density, we obtain

(59) supP:E[X2]≤A−(1+ρ)∫x∈Rln∫y∈Rw(y|x)11+ρfR(y)ρ1+ρdydP(x)(60)=supP:E[X2]≤A−(1+ρ)∫x∈Rln∫y∈R1(2π)11+ρe−(y−x)22(1+ρ)1(2πσ2)ρ1+ρe−y2ρ2σ2(1+ρ)dydP(x)(61)=supP:E[X2]≤A−(1+ρ)∫x∈Rln2π(1+ρ)2ρσ211+ρ12πσ12e−x22σ12dP(x)(62)=supP:E[X2]≤A−(1+ρ)ln(1+ρ)2ρσ211+ρ1σ12+(1+ρ)∫x∈Rx22σ12dP(x)(63)=−(1+ρ)ln(1+ρ)2ρσ211+ρ1σ12+(1+ρ)A2σ12(64)=1+ρ2Aσ12+1+ρ2lnσ12−12lnσ2−1+ρ2ln(1+ρ)2ρ

where in (61) we defined

(65)σ12≜1+ρ+σ2(1+ρ)ρ(66)=Aρβ+(1+ρ)2ρ.

To conclude the proof, it remains to show that the RHS of (64) coincides with $\rho R_{0}\left(\rho \right)$. To this end, observe that some basic algebra reveals that

(67) $$\begin{array}{ccc} \beta \left(\beta -1+\frac{A}{1+\rho }\right) & = & \frac{A}{{\left(1+\rho \right)}^{2}} \end{array}$$

and

(68) $$\begin{array}{ccc} (\beta +\frac{A}{1+\rho })\left(1-\beta \right) & = & \frac{A\rho }{{\left(1+\rho \right)}^{2}}. \end{array}$$

Therefore, the first term in (64) can be rewritten as

(69)1+ρ2Aσ12=1+ρ2AAρβ+(1+ρ)2ρ=1+ρ2Aρ(1+ρ)2βA(1+ρ)2+β(70)=Aρ2(1+ρ)1β+A1+ρ=(1+ρ)(1−β)2,

and the remaining terms rewritten as

1+ρ2lnσ12−12lnσ2−1+ρ2ln(1+ρ)2ρ(71) =1+ρ2lnAρβ+(1+ρ)2ρ−12lnA(1+ρ)β+1−1+ρ2ln(1+ρ)2ρ(72) =1+ρ2lnA(1+ρ)2β+1−12lnA(1+ρ)β+1(73) =ρ2lnβ+A1+ρ+12lnβ.

The sum equals to $\rho R_{0}\left(\rho \right)$.

#### 4.1.2. Lower-Bounding $\lim E_{0}^{(n),*}\left(\rho \right)$

To lower-bound $\lim E_{0}^{(n),*}\left(\rho \right)$, we shall use Proposition 1 with *Q* chosen as a centered variance-A Gaussian distribution $Q_{G}$. For this probability distribution Gallager calculated $E_{0,m}^{*}\left(\rho ,Q_{G}\right)$ (Section 7.4 in [1]). He showed that for any ρ>0,

(74) $$E_{0,m}^{*}\left(\rho ,Q_{G}\right)=\rho R_{0}\left(\rho \right),$$

where $R_{0}\left(\rho \right)$ is defined in (3). Using this result and Proposition 1 we obtain

(75)limn→∞E0(n),*(ρ)≥E0,m*(ρ,QG)(76)=ρR0(ρ).

### 4.2. The Mapping $\rho \mapsto R_{0}\left(\rho \right)$ Is Monotonically Decreasing

For the purpose of proving the achievability of $R_{0}\left(\rho \right)$, we will need the fact that it is monotonically decreasing in ρ. In view of (55), it suffices to show that, for every n∈N, the mapping $\rho \mapsto \rho^{-1}E_{0}^{(n),*}\left(\rho \right)$ is monotonically decreasing. In view of (24), the latter will follow once we establish the monotonicity of $\rho \mapsto \rho^{-1}E_{0}^{\left(n\right)}\left(\rho ,Q^{\left(n\right)}\right)$ for any fixed $Q^{\left(n\right)}$. Since $E_{0}^{\left(n\right)}\left(\rho ,Q^{\left(n\right)}\right)$ evaluates to zero at ρ=0, this monotonicity can be established by showing that the mapping $\rho \mapsto E_{0}^{\left(n\right)}\left(\rho ,Q^{\left(n\right)}\right)$ is concave. This is established in (Appendix 5.B in [1]). (That appendix deals with finite alphabets, but the proof goes through also to our case.)

### 4.3. Achievability of $R_{0}\left(\rho \right)$

The achievability of $R_{0}\left(\rho \right)$ will be proved using a random-coding argument. Let *Q* be the zero-mean variance-A Gaussian distribution, let δ>0 be a positive constant, and let $Q^{\left(n\right)}$ be the distribution on $R^{n}$ defined in (35) and (36). Draw the codewords ${\left\{X_{m}\right\}}_{m=1,\cdots ,e^{nR}}$ of a blocklength-*n* random codebook independently, each according to $Q^{\left(n\right)}$, so $∥X_{m}{∥}^{2}\leq nA$ with probability 1 for every m∈M. By symmetry, $E\left[{\left|L\left(m,Y\right)\right|}^{\rho }\right]$ (where the expectation is over the random choice of codebook and on the channel behavior) does not depend on *m*. Consequently,

(77) $$E\left[e^{-nR}\sum_{m\in M}{\left|L\left(m,Y\right)\right|}^{\rho }\right]=E\left[{\left|L\left(1,Y\right)\right|}^{\rho }\right],$$

and if we establish that $E\left[{\left|L\left(1,Y\right)\right|}^{\rho }\right]$ tends to 1, it will follow by the random-coding argument that there exists a codebook for which the LHS of (77)—with the expectation now over the channel behavior only—tends to 1.

Defining

(78) $$\begin{array}{c} B_{m}\left(x_{1},y\right)=𝟙\left\{w\left(y|X_{m}\right)\geq w\left(y|x_{1}\right)\right\},\;x_{1},y\in R^{n}, \end{array}$$

we can express the RHS of (77) as

(79) $$\begin{array}{ccc} E\left[{\left|L\left(1,Y\right)\right|}^{\rho }\right] & = & E\left[{\left(1+\sum_{m\neq 1}B_{m}\left(X_{1},Y\right)\right)}^{\rho }\right], \end{array}$$

and we seek to show that

(80) $$\begin{array}{c} \lim_{n\to \infty }E\left[{\left(1+\sum_{m\neq 1}B_{m}\left(X_{1},Y\right)\right)}^{\rho }\right]=1. \end{array}$$

To this end, we shall need the following lemma.

**Lemma** **1.** *Let $\left\{Z_{n}\right\}$ be a sequence of random variables taking values in N, and let ρ>0 be fixed. The following two conditions are then equivalent:*

*(i)* 
$E\left[{\left(1+Z_{n}\right)}^{\rho }\right]=1+o\left(1\right)$

*(ii)* 
$E\left[Z_{n}^{\rho }\right]=o\left(1\right)$

*where o(1) tends to zero as n tends to infinity. Thus*

(81) $$\begin{array}{c} \left(\lim_{n\to \infty }E\left[{\left(1+Z_{n}\right)}^{\rho }\right]=1\right)\Leftrightarrow \left(\lim_{n\to \infty }E\left[Z_{n}^{\rho }\right]=0\right). \end{array}$$

**Proof.**  The implication (ii) ⟹ (i) follows by noting for any z∈N and ρ>0

(82) $$\begin{array}{c} {\left(1+z\right)}^{\rho }\leq 1+2^{\rho }z^{\rho }, \end{array}$$

so

(83) $$\begin{array}{ccc} E[{\left(1+Z_{n}\right)}^{\rho }] & \leq & 1+2^{\rho }\;E\left[Z_{n}^{\rho }\right]. \end{array}$$

As for the implication (i) ⟹ (ii), note that any y∈N and ρ>0

(84) $$\begin{array}{c} {\left(1+y\right)}^{\rho }\geq y^{\rho }+𝟙\left\{y=0\right\}, \end{array}$$

so

(85) $$\begin{array}{ccc} E[{\left(1+Z_{n}\right)}^{\rho }] & \geq & E\left[Z_{n}^{\rho }\right]+\mathrm{Pr}\left[Z_{n}=0\right]. \end{array}$$

The implication is now established by noting that (i) implies that $\mathrm{Pr}[Z_{n}=0]\to 1$ because, by Markov’s inequality (and the strict positivity of ρ),

(86)Pr[Zn≠0]=Pr[(1+Zn)ρ−1≥2ρ−1](87)≤E[(1+Zn)ρ]−12ρ−1.

□

In light of the above lemma, to establish (80) it suffices to show that

(88) $$\begin{array}{c} \lim_{n\to \infty }E\left[{\left(\sum_{m\neq 1}B_{m}\left(X_{1},Y\right)\right)}^{\rho }\right]=0, \end{array}$$

i.e., that

(89) $$\begin{array}{c} \lim_{n\to \infty }E\left[E\left[{\left(\sum_{m\neq 1}B_{m}\left(x_{1},y\right)\right)}^{\rho }\;|\;X_{1}=x_{1},Y=y\right]\right]=0, \end{array}$$

where the outer expectation is over $X_{1}$ and Y.

A related expectation—but one where it is the conditional expectation that is raised to the ρ-th power—is studied in the following lemma:

**Lemma** **2.** *If ρ>0 and $R<R_{0}\left(\rho \right)$, then*

(90) $$\begin{array}{c} \lim_{n\to \infty }E\left[E{\left[\sum_{m\neq 1}B_{m}\left(x_{1},y\right)\;|\;X_{1}=x_{1},Y=y\right]}^{\rho }\right]=0. \end{array}$$

**Proof.**  See Appendix A. □

To establish (88) using this lemma, we distinguish between two cases depending on whether 0<ρ≤1 or ρ>1. In the former case $x\mapsto x^{\rho }$ is concave, so Jensen’s inequality implies that

(91) $$\begin{array}{c} E\left[E\left[{\left(\sum_{m\neq 1}B_{m}\left(x_{1},y\right)\right)}^{\rho }\;|\;X_{1}=x_{1},Y=y\right]\right]\leq E\left[E{\left[\sum_{m\neq 1}B_{m}\left(x_{1},y\right)\;|\;X_{1}=x_{1},Y=y\right]}^{\rho }\right], \end{array}$$

which, together with Lemma 2, implies (88) whenever $R<R_{0}\left(\rho \right)$.

Suppose now that ρ>1. Conditional on the transmitted codeword $x_{1}$ and the output y, the random variables ${\left\{B_{m}\right\}}_{m\neq 1}$ are IID Bernoulli, with $B_{m}$ determined by $X_{m}$. We can thus use Rosenthal’s technique (Lemma 5.10 in [19]), [20] to obtain

E∑m≠1Bm(x1,y)ρ|X1=x1,Y=y(92) ≤2ρ2maxE∑m≠1Bm(x1,y)|X1=x1,Y=yρ,E∑m≠1Bm(x1,y)|X1=x1,Y=y(93) ≤2ρ2E∑m≠1Bm(x1,y)|X1=x1,Y=yρ+E∑m≠1Bm(x1,y)|X1=x1,Y=y.

Taking the expectation over $X_{1}$ and Y yields

(94)EE∑m≠1Bm(x1,y)ρ|X1=x1,Y=y(95) ≤2ρ2EE∑m≠1Bm(y)|Y=yρ+2ρ2EE∑m≠1Bm(y)|Y=y(96) ≤2ρ2EE∑m≠1Bm(x1,y)|X1=x1,Y=yρ+2ρ2EE∑m≠1Bm(x1,y)|X1=x1,Y=y.

The first term on the RHS can be treated using the lemma. The second—but for the $2^{\rho^{2}}$ constant—is the one encountered when ρ is 1. Since by Section 4.2, $R_{0}\left(\rho \right)\leq R_{0}\left(1\right)$ (because ρ>1 for the case at hand), it too tends to zero when $R<R_{0}\left(\rho \right)$.

### 4.4. No Rate Exceeding $R_{0}\left(\rho \right)$ Is Achievable

To show the converse, we need Arıkan’s lower bound on guessing [21].

Fix any sequence of rate-*R* blocklength-*n* codebooks $\left\{C_{n}\right\}$ satisfying the cost constraint. For any n∈N, let

(97) $$\begin{array}{c} Q^{\left(n\right)}\left(x\right)=\left\{\begin{array}{cc} \frac{1}{|C_{n}|} & \mathrm{if}\;x\in C_{n},\\ 0 & \mathrm{otherwise} \end{array}\right. \end{array}$$

be the induced probability distribution on $R^{n}$. Since the codebook satisfies the cost constraint, $E[∥X{∥}^{2}]\leq nA$ under $Q^{\left(n\right)}$.

Given y, list the messages m∈M in decreasing order of likelihood $w(y|x_{m})$ (resolving ties arbitrarily, e.g., ranking low numerical values of *m* higher), and let G(m|y) denote the ranking of the message *m* in this list. Note that

(98) $$\begin{array}{c} |L(m,y)|\geq G(m|y), \end{array}$$

where the inequality can be strict because there may be messages that are in L(m,y) because they have the same likelihood as *m*, and that are yet ranked lower than *m* by G(·|y) because of the way ties are resolved. It follows from this inequality that the ρ-th moment of |L(M,Y)| cannot tend to one unless the ρ-th moment of G(M|Y) does. By Arıkan’s guessing inequality [21],

(99) $$\begin{array}{ccc} E\left[G{\left(M|Y\right)}^{\rho }\right] & \geq & {\left(1+nR\right)}^{-\rho }\cdot \exp\left(n\rho R-nE_{0}^{\left(n\right)}\left(\rho ,Q^{\left(n\right)}\right)\right), \end{array}$$

so the ρ-th moment of G(M|Y) can tend to one only if

(100) $$\begin{array}{ccc} \rho R & \leq & \underset{n\to \infty }{lim inf}E_{0}^{\left(n\right)}\left(\rho ,Q^{\left(n\right)}\right). \end{array}$$

From this, the converse now follows using (24) and (55) because

(101)lim infn→∞E0(n)(ρ,Q(n))≤limn→∞E0(n),*(ρ)(102)=ρR0(ρ).

## 5. The Direct Part of Theorem 2

In this section we prove the direct part of Theorem 2: when the decoder can be provided with a rate-$R_{h}$ description of the noise, the convergence (19) can be achieved at all transmission rates below $R_{0}\left(\rho \right)+R_{h}$. As noted earlier, the converse follows directly from (Remark 4 in [3]).

Our proof treats the cases $R_{h}=0$ and $R_{h}>0$ separately. As in Section 4, we assume that the channel is normalized to having noise variance 1 and transmit power A.

### 5.1. Case 1: $R_{h}=0$

The analogous result for the modulo-additive channel was proved in [3] by having the helper provide the decoder with a lossless description of the type of the noise sequence. Since this type fully specifies the a posteriori probability of the transmitted message, the decoder’s remotely-plausible-with-this-help list L(Y,T) contains only messages whose a posteriori probability is equal to that of the correct message. It is therefore a subset of the at-least-as-likely list L(M,Y) (without help) and hence of smaller-or-equal ρ-th moment. Consequently, any rate that allows the latter to tend to one, also allows the former to tend to one.

On the Gaussian channel the likelihood $w(y|x_{m})$ is specified by the normalized squared Euclidean norm of the noise sequence ${∥z∥}^{2}/n$. The latter, however, cannot be described at zero rate with infinite precision. This motivates us to quantize it and have the quantized version be the zero-rate help. The result will then follow by considering the high-resolution limit of the achievable rates. For this purpose, a uniform quantizer will do.

Given some large M>0 (which determines the overload region) and some large *K* (corresponding to the number of quantization cells), we partition the interval [0,M] into *K* subintervals, each of length Δ=M/K. The helper, upon observing the noise sequence Z, produces

(103) $$\begin{array}{c} T=\phi^{\left(n\right)}\left(Z\right)=\left\{\begin{array}{cc} \;\left\lfloor {∥Z∥}^{2}/\left(n\Delta \right)\right\rfloor & {\mathrm{if}\;∥Z∥}^{2}/n<M,\\ \;K & \mathrm{otherwise} \end{array}\right.. \end{array}$$

The constant *M*, which does not depend on the blocklength *n*, is chosen large enough to guarantee that the large-deviation probability of overload $\mathrm{Pr}\left[∥Z{∥}^{2}/n\geq M\right]$ decay sufficiently fast in *n* so that the contribution of the overload to the ρ-th moment of the list be negligible, even if an overload results in the list containing all $e^{nR}$ codewords:

(104) $$\begin{array}{ccc} \lim_{n\to \infty }e^{n\rho R}\cdot \mathrm{Pr}\left[n^{-1}{∥Z∥}^{2}\geq M\right] & = & 0. \end{array}$$

(Upper bounds on the tail of the $\chi^{2}$ distribution show, for example, that for $R<R_{0}\left(\rho \right)$, the choice $M=\max\{2,20\rho R_{0}\left(\rho \right)\}$ will do.) Since the help takes values in the finite set $T_{n}=\left\{0,1,\cdots ,K\right\}$, where *K* does not depend on the blocklength, it is of zero rate.

As in Section 4.3, we consider a random codebook ${\left\{X_{m}\right\}}_{m=1,\cdots ,e^{nR}}$ whose codewords are drawn independently from the conditional Gaussian distribution, i.e., from $Q^{\left(n\right)}$ defined in (35) and (36) with *Q* being $Q_{G}$, the centered variance-A Gaussian distribution. Using the same symmetry arguments, we also assume that the transmitted message is m=1 and study the ρ-th moment of the list under this assumption. Defining

(105) $$\begin{array}{ccc} V_{m}\left(x_{1},y\right) & = & 𝟙\left\{\phi^{\left(n\right)}\left(y-X_{m}\right)=\phi^{\left(n\right)}\left(y-x_{1}\right)\right\},\;x_{1},y\in R^{n}, \end{array}$$

we can express the ρ-th moment of the remotely-plausible list when m=1 as

(106) $$\begin{array}{ccc} E\left[{\left|L\left(Y,T\right)\right|}^{\rho }\right] & = & E\left[{\left(1+\sum_{m\neq 1}V_{m}\left(X_{1},Y\right)\right)}^{\rho }\right]. \end{array}$$

In view of Lemma 1, we need to prove that

(107) $$\begin{array}{c} \lim_{n\to \infty }E\left[{\left(\sum_{m\neq 1}V_{m}\left(X_{1},Y\right)\right)}^{\rho }\right]=0, \end{array}$$

where the expectation is over both the random choice of the codebook and the channel behavior.

To analyze the LHS of (107), we define for every $x_{1},y\in R^{n}$ and every message m≠1 the binary random variable

(108) $$\begin{array}{ccc} B_{m}\left(x_{1},y;\Delta \right) & = & 𝟙\left\{w\left(y|X_{m}\right)\geq w\left(y|x_{1}\right)\cdot e^{-\frac{n\Delta }{2}}\right\}. \end{array}$$

Our analysis of $V_{m}\left(x_{1},y\right)$ depends on whether $\phi^{\left(n\right)}\left(y-x_{1}\right)$ differs from *K* (no overload) or equals *K* (corresponding to quantizer overload). In the former case, the random variable $V_{m}\left(x_{1},y\right)$ can be upper bounded by $B_{m}\left(x_{1},y;\Delta \right)$ because

(109)Vm(x1,y)=𝟙ϕ(n)(y−Xm)=ϕ(n)(y−x1)(110)≤𝟙|∥y−Xm∥2−∥y−x1∥2|<nΔ(111)≤𝟙∥y−Xm∥2≤∥y−x1∥2+nΔ(112)=𝟙e−∥y−Xm∥22≥e−∥y−x1∥2+nΔ2(113)=Bm(x1,y;Δ),

where (110) holds because, for the case at hand, the equality of helper’s description implies that $∥y-X_{m}{∥}^{2}$ and $∥y-x_{1}{∥}^{2}$ lie in a same interval of length nΔ. In the latter case—which is exponentially rare when *M* exceeds the noise variance—we simply upper bound $V_{m}\left(x_{1},y\right)$ by 1.

The ρ-th moment of the list can now be expressed using the law of total expectation as

E∑m≠1Vm(X1,Y)ρ(114) =E∑m≠1Vm(X1,Y)ρ|T≠KPr[T≠K]+E∑m≠1Vm(X1,Y)ρ|T=KPr[T=K](115) ≤E∑m≠1Bm(X1,Y;Δ)ρ|T≠KPr[T≠K]+enρRPr[T=K](116) ≤E∑m≠1Bm(X1,Y;Δ)ρ+enρRPr[T=K].

The second term on the RHS of (116) tends to zero by (104). The first term is studied in the following lemma:

**Lemma** **3.** *If ρ>0, Δ>0, and $R<R_{0}\left(\rho \right)-\Delta$, then*

(117) $$\begin{array}{c} \lim_{n\to \infty }E\left[{\left(\sum_{m\neq 1}B_{m}\left(X_{1},Y;\Delta \right)\right)}^{\rho }\right]=0. \end{array}$$

**Proof.**  See Appendix B. □

For a given $R<R_{0}\left(\rho \right)$, achievability is thus established using this lemma and (116) by picking *M* sufficiently large for (104) to hold, and then picking *K* large enough to guarantee that $R<R_{0}\left(\rho \right)-M/K$ so that, by Lemma 3, the first term on the RHS of (116) will also tend to zero.

### 5.2. Case 2: $R_{h}>0$

The key to proving the achievability of $R_{cutoff}\left(\rho \right)+R_{h}$ is in showing that rate-$R_{h}$ help can be utilized to increase the data rate by $R_{h}$, and that this can be done losslessly, with arbitrarily small (positive) power, and in one channel use. To show how this can be done, we show that—by using the channel once to send a single input that is bounded by $\sqrt{A}$ (with A any prespecified positive number) and using help taking values in the set T={0,…,κ−1}—we can send error-free a message taking values in said set. To transmit m∈{0,…,κ−1}, the encoder sends

(118) $$\begin{array}{c} x=m\cdot \frac{\sqrt{A}}{\kappa }, \end{array}$$

which is upper-bounded by $\sqrt{A}$. Upon observing the noise *Z*, the helper produces the description *T* by quantizing the normalized noise and taking modulo, i.e.,

(119) $$\begin{array}{c} T=\left\lfloor Z\cdot \frac{\kappa }{\sqrt{A}}\right\rfloor \;\mathrm{mod}\;\kappa , \end{array}$$

which is an element of {0,…,κ−1}. Based on *Y* and *T*, the decoder can calculate

(120) $$\begin{array}{c} \hat{m}=\left\lfloor Y\cdot \frac{\kappa }{\sqrt{A}}-T\right\rfloor \;\mathrm{mod}\;\kappa , \end{array}$$

which equals *m*, because

(121)m^=x+Z·κA−Tmodκ(122)=m+Z·κA−Tmodκ(123)=m+Z·κA−Tmodκ(124)=m,

where (123) holds because *m* and *T* are both integers.

Using this building-block, we can now prove the achievability of $R_{cutoff}\left(\rho \right)+R_{h}$ by employing two-phase time sharing. Specifically, we propose the following blocklength-(n+1) scheme. In the first *n* channel uses, the helper operates at rate zero as in Section 5.1. By the achievability result proved in Section 5.1, for any $R<R_{0}\left(\rho \right)$, there exists a sequence of blocklength-*n* rate-*R* codebooks ${\left\{x_{m}\right\}}_{m=1,\cdots ,e^{nR}}$, with $∥x_{m}{∥}^{2}\leq \left(n-1\right)A$ for every *m*, and zero-rate helpers $\phi (Z^{n})$, such that the remotely-plausible-list $L(Y^{n},\phi \left(Z^{n}\right))$ satisfies

(125) $$\begin{array}{c} \lim_{n\to \infty }E\left[|L\left(Y^{n},\phi \left(Z^{n}\right)\right){|}^{\rho }\right]=1. \end{array}$$

In the (n+1)-th channel-use we use the aforementioned coding scheme with κ being $\left\lceil e^{nR_{h}}\right\rceil$. Since that scheme is error-free, the overall remotely-plausible-list for the two phases has the same cardinality as that of the first phase, namely $\left|L(Y^{n},\phi \left(Z^{n}\right))\right|$, and hence, its ρ-th moment tends to 1 by (125).

The achievability now follows by verifying that, the power of the transmitted input sequence x satisfies

(126) $$\begin{array}{c} {∥x∥}^{2}=∥x^{n}{∥}^{2}+{∥x_{n+1}∥}^{2}\leq nA+A=\left(n+1\right)A; \end{array}$$

the rate of the helper is

(127) $$\begin{array}{c} \frac{1}{n+1}\left(0+nR_{h}\right) \end{array}$$

and the rate achieved by the scheme is

(128) $$\begin{array}{c} \frac{1}{n+1}\left(nR_{0}\left(\rho \right)+nR_{h}\right) \end{array}$$

which tend to $R_{h}$ and $R_{0}\left(\rho \right)+R_{h}$, respectively, as *n* tends to infinity.

## Appendix A. Proof of Lemma 2

We shall establish that the expectation

(A1) EE∑m≠1Bm(x1,y)|X1=x1,Y=yρ(A2)=∫y∈Rn∫x1∈RnE∑m≠1Bm(x1,y)|X1=x1,Y=yρw(y|x1)dQ(n)(x1)dν(y)

tends to zero as *n* tends to infinity whenever $R<R_{0}\left(\rho \right)$.

First notice that conditional on the transmitted codeword $x_{1}$ and the channel output y, the random variables ${\left\{B_{m}\right\}}_{m\neq 1}$ are IID Bernoulli, with $B_{m}$ determined by $X_{m}$ and being of probability of success

(A3)p(x1,y)=Prw(y|Xm)≥w(y|x1)(A4)=Prw(y|Xm)11+ρ≥w(y|x1)11+ρ(A5)≤w(y|x1)−11+ρEw(y|Xm)11+ρ,

where the last inequality follows from Markov’s inequality. Thus

EE∑m≠1Bm(x1,y)|X1=x1,Y=yρ(A6) =∫y∈Rn∫x1∈RnE∑m≠1Bm(x1,y)|X=x1,Y=yρw(y|x1)dQ(n)(x1)dν(y)(A7) ≤enρR∫y∈Rn∫x1∈Rnp(x1,y)ρw(y|x1)dQ(n)(x1)dν(y)(A8) ≤enρR∫y∈Rn∫x1∈RnEw(y|Xm)11+ρρw(y|x1)11+ρdQ(n)(x1)dν(y).(A9) =enρR∫y∈RnEw(y|Xm)11+ρρ∫x1∈Rnw(y|x1)11+ρdQ(n)(x1)dν(y)(A10) =enρR∫y∈Rn∫x∈Rnw(y|x)11+ρdQ(n)(x)1+ρdν(y)(A11) ≤erδμ1+ρenρR∫y∈R∫x∈Rer(x2−A)w(y|x)11+ρdQG(x)1+ρdν(y)n(A12) =erδμ1+ρenρRe−nE0,m(ρ,QG,r),

where (A11) follows from the upper bound (39) on the Radon–Nykodim derivative and holds for every r≥0. Choosing *r* as $r^{⋆}$ that achieves $E_{0,m}^{*}\left(\rho ,Q_{G}\right)$ (cf. (31)), we obtain

(A13)EE∑m≠1Bm(x1,y)|X1=x1,Y=yρ≤er⋆δμ1+ρenρRe−nE0,m*(ρ,QG)(A14)=er⋆δμ1+ρenρ(R−R0(ρ)),

where the last equality follows from (74).

The Central Limit Theorem guarantees that, as *n* tends to infinity, μ approaches 1/2. Consequently, the RHS of (A14) tends to zero whenever $R<R_{0}\left(\rho \right)$.

## Appendix B. Proof of Lemma 3

To prove the lemma, we shall establish that, whenever $R<R_{0}\left(\rho \right)-\Delta$,

(A15) $$\begin{array}{c} \lim_{n\to \infty }E\left[E{\left[\sum_{m\neq 1}B_{m}\left(x_{1},y;\Delta \right)\;|\;X_{1}=x_{1},Y=y\right]}^{\rho }\right]=0, \end{array}$$

where the outer expectation is over $X_{1}$ and Y. From this (117) will follow in much the same way that (88) followed from (90) in Section 4.3.

To establish (A15), first note that, conditional on the transmitted codeword $x_{1}$ and the channel output y, the random variables ${\left\{B_{m}\left(x_{1},y;\Delta \right)\right\}}_{m\neq 1}$ are IID Bernoulli, with $B_{m}$ determined by $X_{m}$ and being of probability of success

(A16)p(x1,y;Δ)=Prw(y|Xm)≥w(y|x1)e−nΔ2(A17)=Prw(y|Xm)11+ρ≥w(y|x1)11+ρe−nΔ2(1+ρ)(A18)≤w(y|x1)−11+ρenΔ2(1+ρ)Ew(y|Xm)11+ρ,

where the last inequality follows from Markov’s inequality. Consequently,

EE∑m≠1Bm(x1,y;Δ)|X1=x1,Y=yρ(A19) =∫y∈Rn∫x1∈RnE∑m≠1Bm(x1,y;Δ)|X=x1,Y=yρw(y|x1)dQ(n)(x1)dν(y)(A20) ≤enρR∫y∈Rn∫x1∈Rnp(x1,y;Δ)ρw(y|x1)dQ(n)(x1)dν(y)(A21) ≤enρRenρΔ2(1+ρ)∫y∈Rn∫x1∈RnEw(y|Xm)11+ρρw(y|x1)11+ρdQ(n)(x1)dν(y)(A22) <enρΔenρR∫y∈Rn∫x1∈RnEw(y|Xm)11+ρρw(y|x1)11+ρdQ(n)(x1)dν(y)

where (A22) holds because ρ,Δ>0 so $n\rho \Delta /\left(2(1+\rho )\right)<n\rho \Delta$.

Except for the $e^{n\rho \Delta }$ factor, the RHS of (A22) is identical to the RHS of (A8), which was shown to decay at least as fast as $e^{n\rho (R-R_{0}\left(\rho \right))}$; see (A14). It follows that the RHS of (A22) tends to zero whenever $R+\Delta <R_{0}\left(\rho \right)$.
